# Serum Sphingosine 1-Phosphate (S1P): A Novel Diagnostic Biomarker in Early Acute Ischemic Stroke

**DOI:** 10.3389/fneur.2020.00985

**Published:** 2020-09-08

**Authors:** Jia Liu, Kazuo Sugimoto, Yuanbo Cao, Masahiro Mori, Li Guo, Guojun Tan

**Affiliations:** ^1^Department of Neurology, The Second Hospital of Hebei Medical University, Shijiazhuang, China; ^2^Neurological Laboratory of Hebei Province, Shijiazhuang, China; ^3^Department of Neurology, Dongzhimen Affiliated Hospital, Beijing University of Chinese Medicine, Beijing, China; ^4^Department of Neurology, Graduate School of Medicine, Chiba University, Chiba, Japan

**Keywords:** stroke, ischemic stroke, hemorrhagic stroke, sphingosine 1-phosphate (S1P), high-density lipoprotein cholesterol (HDL-C), biomarker

## Abstract

**Background:** Sphingosine 1-phosphate (S1P) is a lipid metabolite that mediates various physiological processes, including vascular endothelial cell function, inflammation, coagulation/thrombosis, and angiogenesis. As a result, S1P may contribute to the pathogenesis of stroke.

**Objective:** This study aimed to evaluate the diagnostic value of serum S1P in acute stroke.

**Method:** A total of 72 patients with ischemic stroke, 36 patients with hemorrhagic stroke, and 65 controls were enrolled. Serum S1P was detected by enzyme-linked immunosorbent assay (ELISA).

**Results:** Receiver operating characteristic curve analysis demonstrated that serum S1P could discriminate ischemic stroke from hemorrhagic stroke in both total population and subgroup analyses of samples obtained within 24 h of symptom onset (subgroup_ < 24h_) (area under curve, AUC_Total_ = 0.64, *P* = 0.017; AUC_Subgroup < 24h_ = 0.91, *P* < 0.001) and controls (AUC_Total_ = 0.62, *P* = 0.013; AUC_Subgroup <24h_ = 0.83, *P* < 0.001). Furthermore, S1P showed higher efficacy than high-density lipoprotein cholesterol (HDL-C) in discriminating ischemic stroke from controls in the total population (*P*_S1P_ = 0.013, *P*_HDL−C_ = 0.366) and in the subgroup analysis (i.e., <24 h; *P*_S1P_ < 0.001, *P*_HDL−C_ = 0.081). Additionally, lower serum S1P was associated with cervical artery plaques (*P* = 0.021) in controls and with dyslipidemia (*P* = 0.036) and milder neurological impairment evaluated by the National Institute of Health Stroke Scale (NIHSS, *P* = 0.047) in the ischemic stroke group.

**Conclusions:** The present study preliminarily investigated the diagnostic value of serum S1P in acute stroke. Decreased serum S1P may become a potential biomarker for early acute ischemic stroke and can indicate disease severity.

## Introduction

Stroke is a severely disabling neurological disease that encompasses both ischemic and hemorrhagic strokes (1, 2). The pathogenesis and pathophysiological progression of both subtypes are complex and manifest some similarities and differences (1, 3, 4). To diagnose stroke, neuroimaging modalities such as computed tomography (CT) and magnetic resonance imaging (MRI) play an important role in the clinical setting. Biomarkers have also been proven to contribute to diagnosis, to discriminate stroke type, and to identify patients' risk factors, which guide treatment (5, 6). There have been an increasing number of candidate biomarkers for stroke identified in the past decade. Such candidate biomarkers include monitoring biological indicators of coagulation, excitotoxicity, oxidative stress, inflammation, blood–brain barrier dysfunction, and necrosis or apoptosis; however, none have been widely applied in clinical practice (7–9).

Sphingosine 1-phosphate (S1P) is a pleiotropic lipid mediator. Recent studies confirmed red blood cells, endothelial cells, thrombocytes, neutrophils, and macrophages to be the primary sources of S1P in the blood (10, 11). More than 50% of plasma S1P is bound to high-density lipoprotein cholesterol (HDL-C), while about 40% is associated with albumin (12). S1P is a ligand of five G protein-coupled cell surface receptors S1PR1–S1PR5 (13). These receptors are expressed in various cells involving the immune, cardiovascular, respiratory, hepatic, and neurological systems (10, 14). Through differential binding with different receptor subtypes, S1P regulates many physiological and pathological processes, including blood flow, blood pressure, heart rate, vascular endothelial function, atherosclerosis, coagulation/thrombosis, and inflammatory responses, all of which play crucial roles in stroke pathogenesis and progression (10, 13, 15–18).

By activating S1PR1, S1P regulates the egress of lymphocytes from the lymphatic organs into the blood, further regulating the inflammatory process (19). Moreover, the S1P–S1PR1 axis may promote endothelial barrier function and protect endothelial integrity (20). S1P activation of S1PR3 promotes secretion of eNOS-derived NO, protecting cells and tissues from oxidative stress injury (21). The loss of endothelial S1P results in decreased endothelial-derived NO and higher blood pressure (17). Accordingly, fingolimod, an S1P receptor modulator, has been proven to reduce the volume of brain lesions, attenuate neurological impairment, and improve functional outcome in stroke patients by decreasing lymphatic inflammation, regulating cerebrovascular responses, and activating endothelial barrier functions (22–28). In cardiovascular diseases, serum S1P has been reported to show a strong correlation with human coronary artery disease (CAD) onset and severity (29). Another study found that serum S1P concentrations were inversely associated with atherosclerotic diseases, peripheral artery diseases (PAD), and carotid stenosis (CS) in humans (30). However, the clinical relevance of serum S1P in human stroke cases remains unclear. Therefore, in the present study, we aimed to preliminarily evaluate the diagnostic value of serum S1P in acute stroke.

## Methods and Materials

### Participants

In this single-center study at the Second Hospital of Hebei Medical University, patients who were diagnosed with stroke (both ischemic and hemorrhagic strokes) confirmed by CT and/or MRI within 72 h of symptom onset at the Department of Neurology were enrolled in this study. We excluded patients with the following conditions: acute myocardial infarction; any history of a central nervous system condition, such as Parkinson's disease, dementia, or trauma; the presence of other prior systemic diseases, including autoimmune disorder, chronic heart disease, liver cirrhosis, uremia, malignancy, and any kind of infection. Moreover, patients in this study were all acute treatment naïve and thus had received no prior intravenous thrombosis or vascular interventional therapy. The controls were enrolled at the medical examination center of the Second Hospital of Hebei Medical University with no symptom or history of stroke and none of the exclusion criteria described above. Enrollment occurred from 2016 to 2019. In total, we included 72 patients with ischemic stroke, 36 patients with hemorrhagic stroke, and 65 controls. The study was approved by the local ethics committee at the Second Hospital of Hebei Medical University, and written informed consent was obtained from each participant or his/her legal guardian.

The following demographic and clinical data were collected during the first 6 h after admission: gender; age; history of conventional vascular risk factors (e.g., smoking habit, alcohol abuse, history of hypertension, diabetes mellitus, and dyslipidemia); medication at sampling including the application of any anticoagulant, antiplatelet, or statin treatment; and severity of stroke based on the National Institute of Health Stroke Scale (NIHSS). Moreover, patients with ischemic stroke were classified into large-artery atherosclerosis (LAA) or small-vessel occlusion (SAO) groups according to the TOAST classification (31) and were also divided into anterior circulation infarct (ACI) or posterior circulation infarct (POCI) groups according to neuroimaging (CT or MRI) findings.

### Samples Collection

Blood samples were collected in tubes with a coagulating agent within 6 h of patients' hospital admission. The samples were then centrifuged at 3,000 g for 10 min after 2 h of blood sampling to obtain serum, which was immediately frozen and stored at −80°C until further analysis. The serum was tested for the following components using the biochemical method in the Clinical Test Laboratory of the Second Hospital of Hebei Medical University: total cholesterol (mmol/L), triglyceride (mmol/L), low-density lipoprotein cholesterol (LDL-C) (mmol/L), and HDL-C (mmol/L).

### S1P Measurement

Serum S1P concentrations were measured with a commercially available ELISA kit (Wuhan USCN Business Co., Ltd., Hubei, China) following the manufacturer's instructions. In brief, serum S1P, co-incubated with a solid-phase human monoclonal antibody was detected with a biotin-labeled polyclonal antibody. A streptavidin–peroxidase conjugate was then added to bind the biotinylated antibody. After that, a tetramethylbenzidine substrate was added, and the mixture was measured at 450 nm. The concentration of S1P in the serum samples was determined by comparison to the standard curve.

### Statistical Analysis

A Kruskal–Wallis test with Bonferroni adjustment for multiple comparisons was used to compare three or more groups, Mann–Whitney *U*-test was used for the comparisons between two groups, and Spearman's rank correlation was used for the correlation analysis between indexes. We used receiver operating characteristic (ROC) curves to evaluate discriminatory power between the ischemic and hemorrhagic stroke groups, as well as controls. The Youden index (sensitivity + specificity − 1) was calculated to determine the cutoff value, which maximizes discriminating accuracy. Furthermore, to evaluate and adjust for the influence of patients' demographic features and underlying conditions (smoking habit, alcohol abuse, hypertension, diabetes mellitus, and dyslipidemia), univariate analysis with a general linear model was employed, setting the demographic features and underlying conditions as the covariate. Fisher's exact test was used to test all categorical data. Values of *P* < 0.05 were considered as statistically significant. Statistical analysis was performed using SPSS 21.0 software.

## Results

### Patient Demographic and Clinical Characteristics

Demographic and clinical characteristics of disease groups and controls are listed in Table 1. There was no significant difference in age at sampling among the three groups, while the ischemic stroke group had a higher proportion of male patients than the controls (*P* = 0.036). As for the conventional vascular risk factors (see Methods and Materials), the ischemic stroke group showed a higher proportion of patients with a smoking habit compared with the controls (*P* < 0.01), as well as a higher proportion of patients with alcohol abuse compared with both control (*P* < 0.01) and hemorrhagic stroke groups (*P* = 0.025). The hemorrhagic stroke group contained a higher proportion of patients with hypertension compared with controls (*P* < 0.01) and a higher proportion of patients with diabetes mellitus compared with both the ischemic stroke (*P* = 0.014) and control groups (*P* < 0.01).

**Table 1:** The demographic and clinical characteristics of stroke patients and controls.

	**IS**	**HS**	**Con**	***P*****-value**^**$**^		
				**IS vs. HS**	**IS vs. Con**	**HS vs. Con**
**Number**	72	36	65	72 vs. 36	72 vs. 65	36 vs. 65
**Demographic data**						
Sex (F/M)	23/49	15/21	33/32	0.393	**0.036**	0.412
Age (years)^†^	58.7 ± 9.7	55.5 ± 12.4	56.6 ± 8.5	0.398	0.126	0.868
Sampling duration (days)^†^	1.6 ± 1.0	1.3 ± 1.1	n/a	0.164	n/a	n/a
Smoking habit (Y/N)	24/48	6/27	3/62	0.162	**<0.01**	0.057
Alcohol abuse (Y/N)	21/51	3/30	5/60	**0.025**	**<0.01**	1
**Clinical parameters**						
History of hypertension (Y/N)	47/25	29/7	33/32	0.125	0.118	**<0.01**
History of diabetes mellitus (Y/N)	22/50	3/33	26/39	**0.014**	0.284	**<0.01**
History of dyslipidemia	12/60	7/29	5/60	0.791	0.127	0.109
Low-density lipoprotein cholesterol (LDL-C) (mmol/L)^†^	2.7 ± 0.8	2.3 ± 0.9	1.3 ± 0.5	**<0.01**	0.276	**<0.01**
High-density lipoprotein cholesterol (HDL-C) (mmol/L)^†^	1.0 ± 0.2	1.2 ± 0.3	3.5 ± 0.8	**<0.01**	0.397	**<0.01**
Triglyceride (mmol/L)^†^	1.8 ± 1.8	1.6 ± 0.9	1.3 ± 1.1	1	1	0.369
Total cholesterol (mmol/L)^†^	5.3 ± 0.9	4.2 ± 1.1	3.9 ± 1.1	**<0.01**	**<0.01**	1
History of previous stroke (Y/N)	17/55	1/35	0/65	**<0.01**	**<0.01**	1
Cervical artery plaques (Y/N)	72/0	n/a	26/18	n/a	**<0.01**	n/a
**Medication at sampling**						
ASP/CLO (Y/N)	20/52	0/36	4/61	**<0.01**	**0.033**	0.294
Statins (Y/N)	18/54	0/36	4/61	**<0.01**	**<0.01**	0.294

IS, ischemic stroke; HS, hemorrhagic stroke; Con, controls; ASA, aspirin; CLO, clopidogrel; n/a, not available.

Bold type indicates a significant difference.

Mean ± standard deviation.

$ *ruskal–Wallis test with Bonferroni adjustment for multiple comparisons was used to compare the demographic data or clinical parameters among three groups. Mann–Whitney U-test was used for the comparison of the demographic data or clinical parameters between two groups. Fisher's exact test was used to test all categorical data*.

Additionally, patients with ischemic stroke showed a higher level of total cholesterol than did either the hemorrhagic stroke (*P* < 0.01) or control group (*P* < 0.01). Although there was a similar proportion of subjects with a history of dyslipidemia among all groups, patients with either ischemic or hemorrhagic stroke showed both a higher level of LDL-C (*P*_Ischemic_ < 0.01, *P*_Hemorrhagic_ < 0.01) and lower HDL-C levels than the controls did (*P*_Ischemic_ < 0.01, *P*_Hemorrhagic_ < 0.01) at sampling. There was a higher proportion of ischemic stroke patients who had a history of previous stroke compared to hemorrhagic stroke and controls (*P*_Ischemic−controls_ < 0.01, *P*_Ischemic−Hemorrhagic_ < 0.01). In terms of medication at sampling, a higher proportion of patients with ischemic stroke were treated with aspirin/clopidogrel (ASP/CLO) or a statin compared to patients with hemorrhagic stroke (*P*_ASP/CLO_ < 0.01, *P*_Statin_ < 0.01) and controls (*P*_ASP/CLO_ = 0.033, *P*_Statin_ < 0.01).

### Serum S1P Level in Stroke

**Figure 1:**
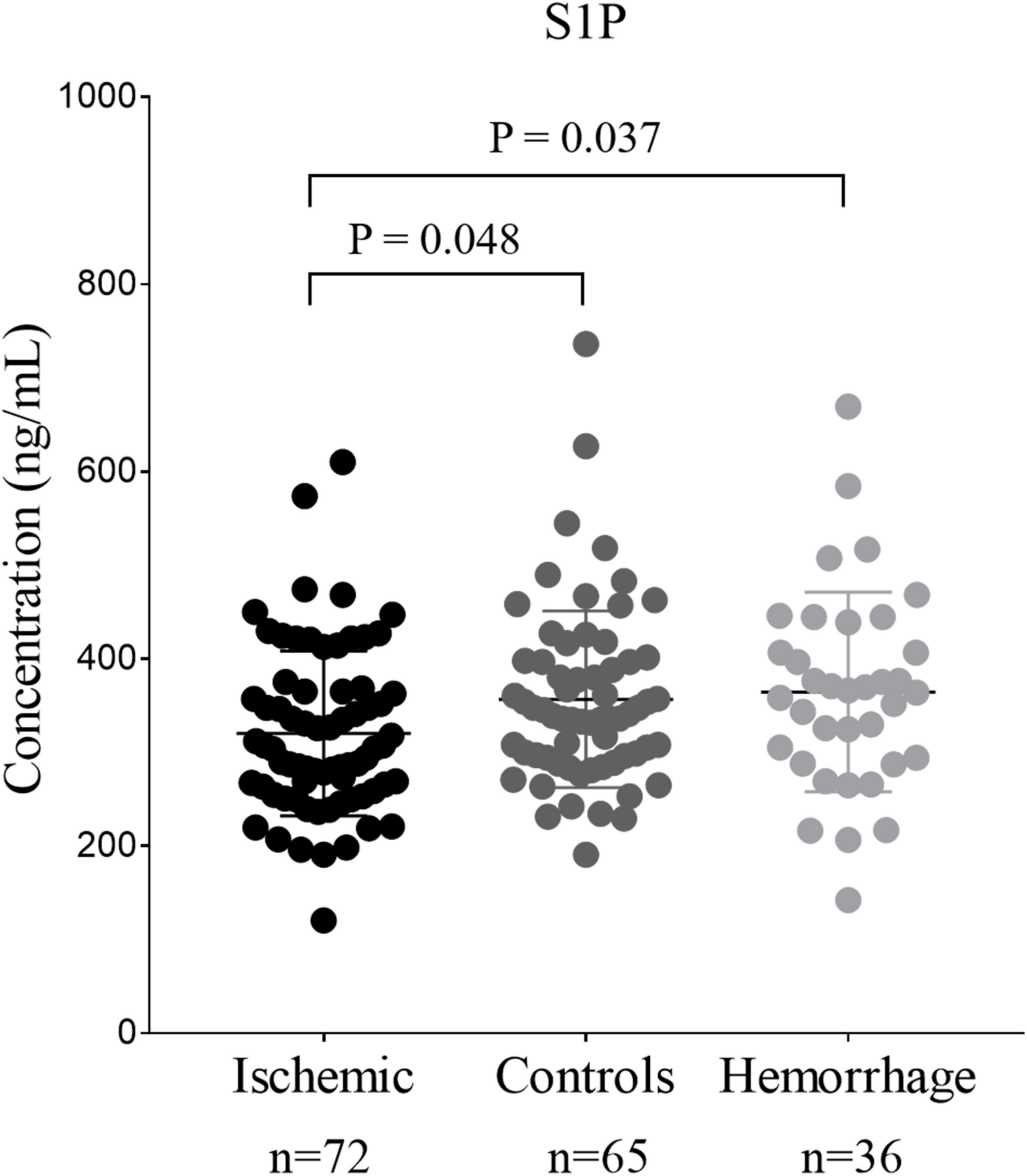
The comparison of serum sphingosine 1-phosphate (S1P) concentration among patients with stroke and controls. Dots represent the serum S1P concentration in ischemic stroke, hemorrhage stroke, and controls. Bars represent mean ± SD (standard deviation). Ischemic, ischemic stroke; Hemorrhage, hemorrhagic stroke.

**Figure 2:**
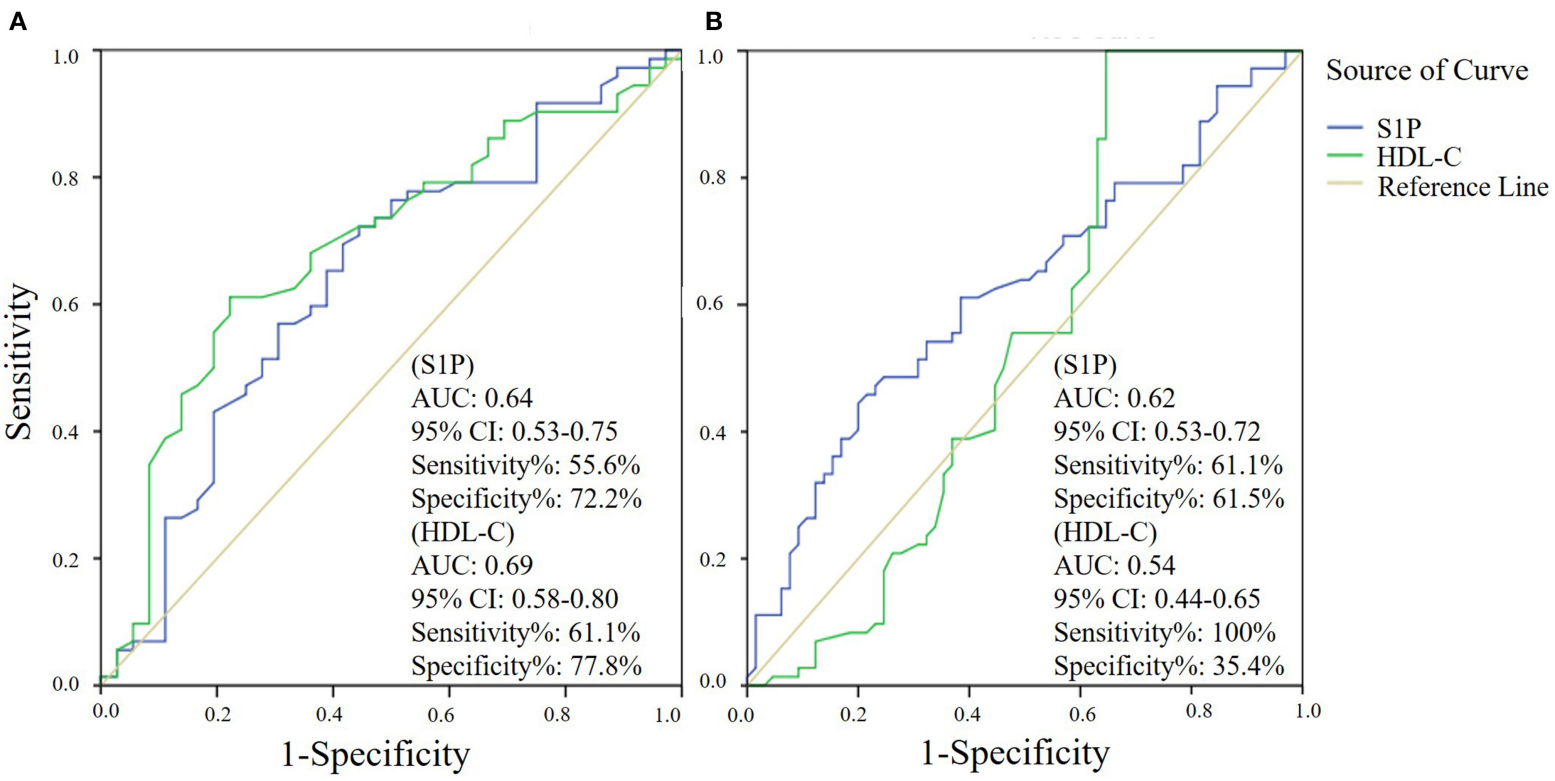
Serum sphingosine 1-phosphate (S1P) and high-density lipoprotein cholesterol (HDL-C) levels as diagnostic biomarkers for ischemic stroke by receiver operator characteristic (ROC) curve analysis. ROC curve analysis of the diagnostic value of serum S1P and HDL-C in discriminating ischemic stroke and hemorrhagic stroke **(A)** and in discriminating ischemic stroke and controls **(B)**.

**Figure 3:**
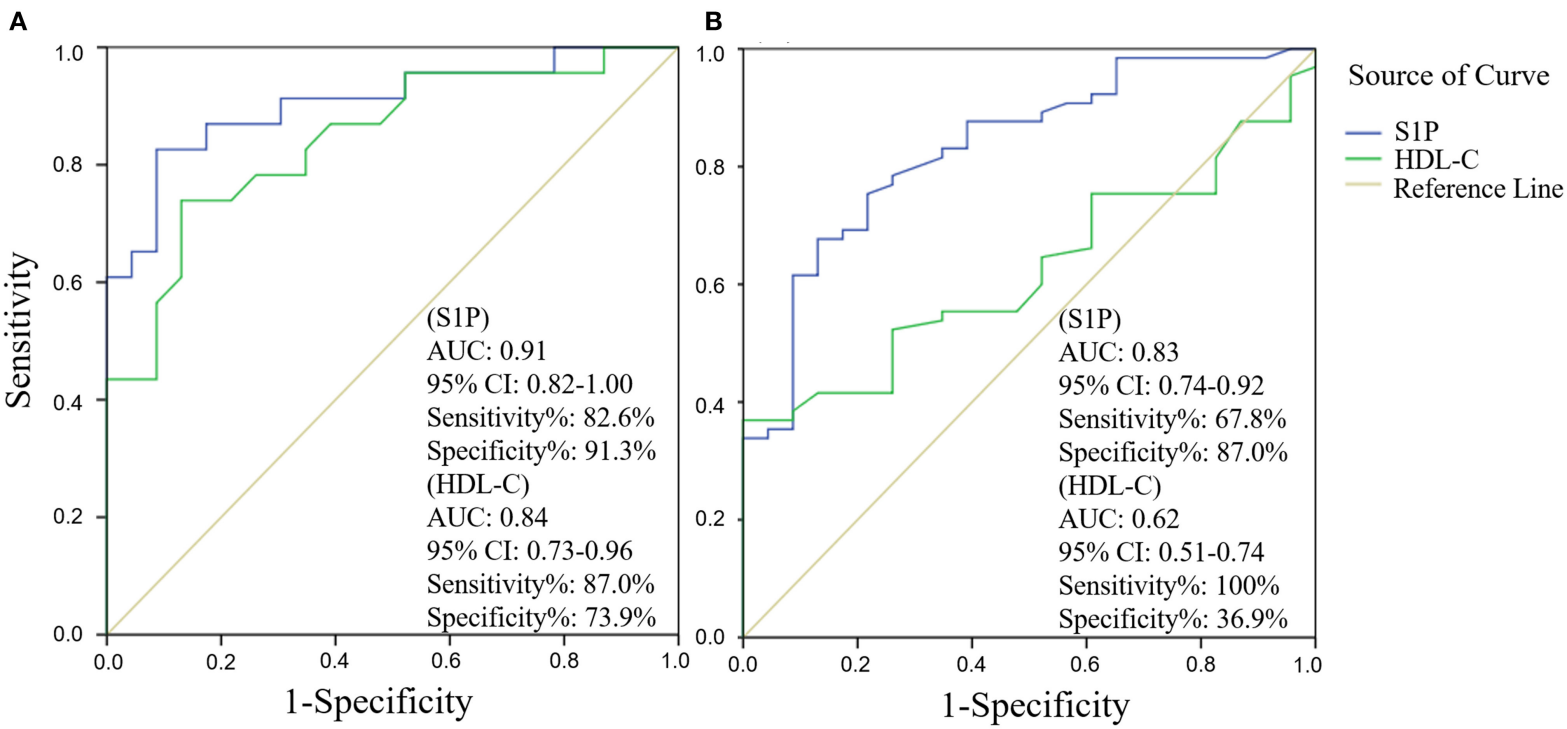
Subgroup analysis (sampling within 24 h after symptom onset) of serum sphingosine 1-phosphate (S1P) and high-density lipoprotein cholesterol (HDL-C) levels as diagnostic biomarkers for ischemic stroke by receiver operator characteristic (ROC) curve. ROC curve analysis of the diagnostic value of serum S1P and HDL-C in discriminating ischemic stroke from hemorrhagic stroke **(A)** and in discriminating ischemic stroke from controls **(B)**.

The comparison of serum S1P concentration between stroke patients and controls is shown in Figure 1. Patients with ischemic stroke showed lower serum S1P concentration compared with hemorrhagic stroke patients [mean ± standard deviation (SD): 320.2 ± 88.1 vs. 364.4 ± 106.6 ng/ml; *P* = 0.037] and controls (mean ± SD: 320.2 ± 88.1 vs. 356.7 ± 94.4 ng/ml; *P* = 0.048). Based on ROC curve analysis (Figure 2), the optimal cut-off value of serum S1P concentration for discriminating ischemic stroke patients from controls was 331.3 ng/ml, and the cutoff value to discriminate ischemic stroke from hemorrhagic stroke was 357.8 ng/ml; the area under the curve (AUC) was 0.62 when comparing ischemic stroke patients and controls and 0.64 when comparing ischemic and hemorrhagic stroke patients, which yielded sensitivities of 61.1 and 55.6% and specificities of 61.5 and 72.2%, respectively. We further divided patients with stroke according to sampling time after symptom onset into the following three subgroups: <24 h (*n*_IS_ = 23; *n*_HS_ = 23), 24–48 h (*n*_IS_ = 29; *n*_HS_ = 5), and 48–72 h (*n*_IS_ = 20; *n*_HS_ = 8) (IS = ischemic stroke, HS = hemorrhagic stroke). The concentrations of serum S1P in the <24, 24–48, and 48–72 h subgroups were (mean ± SD) 258.9 ± 57.7, 357.0 ± 94.5, and 337.3 ± 71.0 ng/ml, respectively, as well as 397.8 ± 102.9, 358.2 ± 73.3, and 272.3 ± 84.1 ng/ml in the corresponding hemorrhagic stroke group. Next, we compared the serum S1P concentration at different sampling time points within the ischemic and hemorrhagic groups. The <24 h subgroup showed decreased serum S1P levels compared to the 24–48 h (*P* < 0.001) and 48–72 h (*P* = 0.005) subgroups in ischemic stroke patients. In hemorrhagic stroke patients, the 48–72 h subgroup showed decreased serum S1P levels compared to the <24 h subgroup (*P* = 0.012), but the serum S1P levels were not significantly different from those of the 24–48 h subgroup (*P* = 0.37). The results of serum S1P level as a diagnostic biomarker for ischemic stroke when sampled within 24 h are shown in Figure 3. Based on ROC curve analysis, the optimal cutoff value of S1P was 307.7 ng/ml for discriminating ischemic stroke from controls and 321.3 ng/ml for discriminating ischemic stroke from hemorrhagic stroke. The AUCs for discriminating ischemic from hemorrhagic stroke and discriminating ischemic stroke from controls were 0.91 and 0.83, respectively, which yielded respective sensitivities of 82.6% and 67.8% and specificities of 91.3 and 87.0%. However, in the 24–48 and 48–72 h subgroups, there was no significant difference in serum S1P levels among groups (*P* > 0.05).

Next, we evaluated the influence of demographic features and underlying conditions including gender (age was not set as a covariate, as there was no significant difference in age among groups), hypertension, diabetes mellitus, dyslipidemia, smoking habit, and alcohol abuse on the comparison of serum S1P levels among groups using a general linear model. The results showed that the above factors had no significant influence on serum S1P level comparison, despite gender (*P* = 0.009). After adjusting for gender, the results still showed a significant difference in serum S1P concentration between ischemic stroke and control groups (*P* = 0.033) and between ischemic and hemorrhagic stroke groups (*P* = 0.024). In addition, we compared the accuracy of serum S1P and HDL-C in discriminating stroke from controls and different stroke types (Figures 2, 3). Decreased serum S1P and HDL-C concentrations possessed similar accuracy in discriminating ischemic stroke from hemorrhagic stroke (AUC_S1P_ = 0.64, *P* = 0.017; AUC_HDL−C_ = 0.69, *P* = 0.001), while decreased serum S1P concentration manifested better accuracy in discriminating ischemic stroke from controls (AUC_S1P_ = 0.62, *P* = 0.013; AUC_HDL−C_ = 0.54, *P* = 0.366). Moreover, in the <24 h subgroup, we found that decreased serum S1P concentration manifested similar accuracy to decreased HDL-C concentration in discriminating ischemic stroke from hemorrhagic stroke (AUC_S1P_ = 0.91, *P* < 0.001; AUC_HDL−C_ = 0.84, *P* < 0.001), while decreased serum S1P concentration manifested superior accuracy to HDL-C in discriminating ischemic stroke from controls (AUC_S1P_ = 0.83, *P* < 0.001; AUC_HDL−C_ = 0.62, *P* = 0.081).

The relationship between serum S1P levels and conventional vascular risk factors in stroke patients and controls is shown in Tables 2–4. Control group subjects with cervical artery plaques had lower serum S1P levels than ones without plaques (*P* = 0.021). In the ischemic stroke group, patients with dyslipidemia manifested lower serum S1P levels than patients without dyslipidemia (*P* = 0.036). Patients with severe neurological disability due to stroke showed higher serum S1P levels than patients with milder disability as evaluated by the NIHSS score (*P* = 0.047). However, serum S1P level showed no significant difference in ischemic subgroups divided by either etiology (*P* = 0.252) or tomography analysis (*P* = 0.101). Furthermore, there was no significant difference in serum S1P levels for patients with or without a history of stroke in the ischemic stroke group (*P* = 0.614). As for the influence of medication on serum S1P concentration, patients treated with ASP/CLO or statins showed similar serum S1P concentrations to patients without medication (*P*_ASA_ = 0.597; *P*_Statin_ = 0.499). The hemorrhagic stroke group showed no significant difference in serum S1P concentration between subgroups divided by clinical parameters.

**Table 2:** Association of serum S1P levels (ng/ml) with conventional vascular risk factors in patients with hemorrhagic stroke.

**Risk factors**	**Frequency (%)**	**S1P (without) (mean ± SD)**	**S1P (with) (mean ± SD)**	***P*-value^**$**^**
Male	32 (49.2%)	364.9 ± 114.5	348.3 ± 68.6	0.953
Age (≥55)	36 (55.4%)	354.9 ± 85.2	358.1 ± 102.4	0.822
History of hypertension	29 (50.8%)	377.7 ± 121.0	336.4 ± 52.8	0.435
History of diabetes mellitus	26 (40.0%)	356.7 ± 93.8	356.7 ± 97.2	0.888
Dyslipidemia	26 (40.0%)	355.7 ± 99.3	357.4 ± 92.3	0.713
Smoking habits	3 (4.6%)	355.0 ± 94.8	392.1 ± 96.2	0.444
Alcohol abuse	5 (7.7%)	355.2 ± 96.3	374.6 ± 72.7	0.453
Cervical artery plaques	26/44 (59.1%)	390.9 ± 106.0	319.3 ± 67.2	**0.021**

Bold type indicates a significant difference.

$ *Mann–Whitney U-test*.

**Table 3:** Association of serum S1P levels (ng/ml) with conventional vascular risk factors in patients with hemorrhage stroke.

**Risk factors**	**Frequency (%)**	**S1P (without) (mean ± SD)**	**S1P (with) (mean ± SD)**	***P*-value^**$**^**
Male	21 (58.3%)	411.5 ± 111.8	330.8 ± 91.0	0.067
Age (≥55)	20 (55.6%)	345.0 ± 114.3	380.0 ± 100.2	0.445
History of hypertension	29 (80.6%)	337.9 ± 75.8	370.8 ± 112.9	0.535
History of diabetes mellitus	3 (8.3%)	370.4 ± 109.2	298.4 ± 30.4	0.115
Dyslipidemia	13 (36.1%)	388.8 ± 117.8	334.3 ± 113.4	0.219
Smoking habits	6 (16.7%)	354.3 ± 101.6	402.3 ± 156.4	0.225
Alcohol abuse	3 (8.3%)	363.7 ± 114.4	356.2 ± 89.3	0.925
Cervical artery plaques	n/a	n/a	n/a	n/a

n/a, not available.

$ *Mann–Whitney U-test*.

**Table 4:** Association of serum S1P levels (ng/ml) with conventional vascular risk factors, clinical parameters, and medication in patients with ischemic stroke.

**Risk factors**	**Frequency (%)**	**S1P (without) (mean ± SD)**	**S1P (with) (mean ± SD)**	***P*-value^**$**^**
Male	49 (68.1%)	319.9 ± 87.4	320.3 ± 89.3	0.708
Age (≥55)	47 (65.3%)	339.5 ± 102.3	309.9 ± 78.7	0.295
History of hypertension	47 (65.3%)	340.3 ± 99.2	309.5 ± 80.6	0.284
History of diabetes mellitus	22 (30.6%)	314.9 ± 93.4	332.2 ± 75.0	0.271
Dyslipidemia	54 (75.0%)	337.6 ± 89.5	282.1 ± 78.0	**0.036**
Smoking habits	24 (33.3%)	310.1 ± 73.2	340.2 ± 111.2	0.448
Alcohol abuse	21 (7.7%)	313.6 ± 77.3	336.1 ± 110.5	0.532
Cervical artery plaques	72 (100%)	n/a	320.2 ± 88.1	n/a
**Clinical parameters**				
History of previous stroke	17 (23.6%)	319.9 ± 89.5	320.9 ± 85.9	0.614
NIHSS ≥ 5	26 (36.1%)	305.4 ± 82.0	346.2 ± 93.9	**0.047**
ACI vs. (POCI)	56 (77.8%)	290.7 ± 72.3	329.6 ± 93.2	0.252
LAA vs. (SAO)	36 (50.0%)	305.3 ± 87.5	335.0 ± 87.2	0.101
**Medication**				
ASP/CLO	20 (27.8%)	318.9 ± 92.1	323.4 ± 78.6	0.597
Statin	18 (25.0%)	318.4 ± 92.8	325.3 ± 74.1	0.499

ACI, anterior circulation infarcts; POCI, posterior circulation infarcts; LAA, large-artery atherosclerosis; SAO, small-vessel occlusion; NIHSS, National Institute of Health Stroke Scale; ASA, aspirin; CLO, clopidogrel; and n/a, not available.

Bold type indicates a significant difference.

$ *Mann–Whitney U-test*.

## Discussion

In the present study, we revealed that decreased serum S1P concentration could discriminate ischemic stroke from hemorrhagic stroke and controls and that lower serum S1P levels were associated with cervical artery plaques in controls and with dyslipidemia and milder neurological impairment in ischemic stroke patients. Furthermore, in the <24 h subgroup, serum S1P levels showed better sensitivity and specificity in discriminating ischemic stroke from controls than it showed for the total enrolled patient population. As for serum S1P detection, the mass spectrometry or liquid chromatography coupled to tandem mass spectrometry (LC-MS/MS) was widely applied in clinical or preclinical studies with high sensitivity and accuracy. Moreover, by applying this technology, the HDL-associated S1P, LDL-associated S1P, and albumin-associated S1P could also be detected quantitatively. These were proven to play differentiating roles in different physiological processes (30, 32, 33). The methodology of serum S1P detection by ELISA has been established and employed by several studies (34–36). Moreover, in the present study, ELISA determined that the concentration of serum S1P in controls was 356.7 ± 94.4 ng/ml (0.94 ± 0.25 nmol/ml), showing a similar range as was found in previous reports of healthy groups (1.04 ± 0.24 and 0.88 ± 0.21 nmol/ml) using the mass spectrometry or LC-MS/MS (30, 33), which may verify the reliability of our detection system and the accuracy of our serum S1P data.

S1P regulates a wide array of biological functions in endothelial cells, including mediation of pro-inflammatory responses and maintenance of barrier function and integrity (13). Intercranial atherosclerotic disease is a highly prevalent cause of stroke (37). The central feature of that is a dysfunctional vascular endothelial barrier, which leads to increased vascular permeability, which in turn causes the decreased concentration of serum S1P, as the endothelium is an important serum S1P source (38, 39). Decreased S1P levels will further promote endothelial inflammation by upregulating pro-inflammatory endothelial markers such as vascular cell adhesion molecule 1 (VCAM-1) and intercellular adhesion molecule 1 (ICAM-1), inducing reorganization of the actin cytoskeleton and regulating endothelial adherent junctions, eventually exacerbating the damage to the endothelial cell barrier (40, 41). Furthermore, with the occurrence of stroke, hypoxic ischemia-induced mitochondrial dysfunction, excitotoxicity, and oxidative stress damage further aggravate the destruction of vascular endothelial cells, leading to impaired function and integrity of the endothelial barrier (37, 42). Therefore, under pathological conditions, disturbed homeostasis of serum S1P levels and S1P signaling dysfunction will promote the process of adverse vascular events such as stroke. Accordingly, fingolimod, an S1P analog, was proven to attenuate neurological impairment not only in the animal model but also in patients with acute stroke, through regulating endothelial adherent junctions, cerebrovascular responses, and blood–brain barrier functions by functional activation of S1P signaling (22, 26).

In addition, S1P is believed to play a pivotal role in regulating lymphocyte egress from the thymus or secondary lymphoid organs to circulation, the major factor of which is the S1P gradient. S1P levels are lower in intracellular and interstitial fluid, whereas S1P is enriched within the blood and lymph. Also, disruption of the S1P gradient results in lymphopenia due to a defect in lymphocyte egress (43). In the present study, decreased serum S1P may lead to lymphopenia through the aforementioned mechanisms, the effect of which was similar to the immunomodulation of fingolimod in stroke patients. The latter was proven to efficiently reduce neurological disability in ischemic stroke patients by redistributing lymphocytes from the circulation to secondary lymphoid organs, which subsequently causes lymphopenia (22, 23, 25). Moreover, a recent report further showed that a certain period of lymphopenia (>24 h) is an efficient measure against ischemic stroke (44). In our study, the significantly declined level of serum S1P was especially found within 24 h of symptom onset for the ischemic stroke patients. Based on the above, the lymphopenia caused by decreased serum S1P in the ischemic stroke patients revealed in this study may be weak and may not be sustained. Additionally, S1P signaling was also shown to be associated with other immunological processes involving T-cell activation status, tumor necrosis factor (TNF)-mediated inflammation, and dendritic cell differentiation (45). Therefore, the relationship between the serum S1P level and its role in immunomodulation and post-stroke immunosuppression needs to be investigated further by *in vitro* and *in vivo* studies.

In the present study, we found that decreased serum S1P levels were associated with ischemic stroke, which was similar to previous findings that PAD and CS are associated with decreased serum S1P concentrations (30). In addition, we found that serum S1P concentration also decreased in patients with ischemic stroke compared to patients with hemorrhagic stroke. This may be due to the different pathological processes of these two types of stroke and differential medical treatment. After a hemorrhagic stroke, the injured vascular system promptly activates thrombin within a few hours (4), independent of serum S1P secretion (46). The activated thrombin and platelets themselves as well as the NF-κB signaling pathway, upregulated by the protease-activated receptor (PAR), may indirectly elevate the serum S1P levels (46). Of note, fingolimod treatment was shown to lead to an obvious amelioration of brain tissue loss in both intracerebral hemorrhage and subarachnoid hemorrhage models and patients through reducing lymphocytic inflammation, preserving the blood–brain barrier, and limiting brain edema by functional activation of S1P signaling (24, 27, 28).

Different prescribed medications were considered to be another influential factor for the discrepancy in serum S1P concentrations. Aspirin and statins are taken by many patients with ischemic stroke, and less frequently by patients with hemorrhagic stroke or controls. Previous studies showed that aspirin could block the PAR-1-stimulated release of S1P in platelets *ex vivo* (47), while statins have been reported to cause no significant increase in plasma S1P (48). However, in the present study, the serum concentration of S1P showed no significant difference between patients with aspirin or statin treatment and patients without treatment. Moreover, the decreased serum S1P level in the sample taken within 24 h after symptom onset from ischemic stroke patients who were almost without any medication showed more power to discriminate ischemic stroke from hemorrhagic stroke, as well as from controls, than it showed in the total patient population. Taken together, based on our study and previously published work, it is unclear whether aspirin and statins are major determinants of serum S1P concentration, and this needs to be clarified in future studies (30).

CT and MRI have been widely applied in the clinical setting for decades to diagnose stroke. However, for patients with early acute or hyperacute stroke, the diagnostic accuracy of CT for ischemic stroke is <30%; although the diagnostic accuracy of MRI is ~70–90%, it has the disadvantages of a long inspection time, comparatively high cost, and lack of widespread application (49). Therefore, serum S1P, which shows higher sensitivity and specificity to identify ischemic stroke, especially within 24 h of symptom onset, appears to be a potential auxiliary indicator for early ischemic stroke diagnosis. Of note, more than 50% of plasma S1P is bound to HDL-C. This may raise doubts as to whether HDL-C and S1P worked independently or whether HDL-C worked through S1P. In the present study, we revealed differential discrimination of these factors among stroke patients and controls, which indicates the independent but synergistic action of these two markers.

In the control group, we found that decreased serum S1P concentration was associated with cervical artery plaques. In ischemic stroke, lower serum S1P concentration was associated with dyslipidemia, which suggests that abnormal levels of serum S1P contribute to abnormal lipid metabolism and further promote the formation of plaques and atherosclerosis (13). Additionally, in the ischemic stroke group, patients with severe neurological disability showed higher serum S1P level than patients with a milder disability as evaluated by the NIHSS score. Severe neurological disability may result from extensive cerebral infarction caused by blockage of the intracranial aorta, which may lead to a compensatory increase in serum S1P to regulate factors such as coagulation and platelet action that lead to thrombosis (50). Further study with larger cohorts is needed to confirm this and to determine the exact mechanism, requiring investigation in related *in vivo* and *ex vivo* studies.

Overall, our study still has some limitations. First, the sample number of both patients and controls was limited, which makes it difficult to conduct comprehensive statistical analysis among subgroups and make determined conclusions. Second, we lack long-term follow-up for individual patients. The lack of long-term follow-up prevents us from monitoring the fluctuations of serum S1P levels with disease progression, as well as treatment, and thus limited our ability to determine the predictive value of serum S1P for disease prognosis. Third, serum S1P was detected by ELISA rather than the more sensitive mass spectrometry or LC–MS/MS technology, and detailed analysis of the level of HDL-associated S1P, LDL-associated S1P, and albumin-associated S1P was lacking. Fourth, this study lacked sufficient data obtained from neuroimaging (CT/MRI) and further analysis of its association with serum S1P level. Therefore, the conclusions of the present study should be considered with caution. Further longitudinal studies with a greater number of patients, employing more sensitive and accurate detection technology, with adequate neuroimaging data are needed to confirm the present study's findings.

## Conclusions

The present study preliminarily investigated the diagnostic value of serum S1P in acute stroke. Decrease in serum S1P may become a potential biomarker for early acute ischemic stroke and can indicate disease severity.

## Data Availability Statement

The raw data supporting the conclusions of this article will be made available by the authors, without undue reservation.

## Ethics Statement

The studies involving human participants were reviewed and approved by Research Ethics Committee of the second hospital of Hebei Medical University. The patients/participants provided their written informed consent to participate in this study.

## Author Contributions

JL and KS contributed to the conception and design, acquisition, analysis and interpretation of data, statistical analysis, and drafting and revision of the manuscript. YC contributed to the conception and design, analysis and interpretation of data, and revision of the manuscript. MM contributed to the acquisition of data and revision of the manuscript. LG and GT contributed to study supervision. All authors contributed to the article and approved the submitted version.

## Conflict of Interest

The authors declare that the research was conducted in the absence of any commercial or financial relationships that could be construed as a potential conflict of interest.
